# Neurosarcoidosis Presenting as Longitudinally Extensive Transverse Myelitis and Orbital Mass: A Case Report

**DOI:** 10.7759/cureus.37821

**Published:** 2023-04-19

**Authors:** Nicholas Cassimatis, Ellen Hong, Andrew Trippiedi, Simeon A Lauer

**Affiliations:** 1 Neurological Surgery, Hackensack Meridian School of Medicine, Hackensack, USA; 2 Internal Medicine, Hackensack Meridian School of Medicine, Hackensack, USA; 3 Ophthalmology, Hackensack Meridian School of Medicine, Hackensack, USA; 4 Ophthalmology, Hackensack University Medical Center, Hackensack, USA

**Keywords:** orbital biopsy, granuloma, sarcoidosis, opthalmology, transverse myelitis, spine, neurosarcoidosis

## Abstract

We describe a case of neurosarcoidosis in a 64-year-old female who presented with proptosis and orbital inflammation together with bilateral lower extremity neuropathy and longitudinally extensive transverse myelitis. These two entities are not commonly associated, and the etiology of the transverse myelitis was facilitated by an orbital biopsy. The transverse myelitis caused numbness in her lower extremities and tightness in her chest and abdomen, which progressed over weeks to difficulty walking and bilateral neuromuscular weakness. Magnetic resonance imaging (MRI) revealed longitudinally extensive transverse myelitis in the cervical and thoracic spine. Computed tomography (CT) imaging of the chest revealed right hilar and mediastinal lymphadenopathy and calcified subcarinal nodes. Positron emission tomography (PET) scan revealed hypermetabolism in the mediastinum and medial left orbit. Orbital biopsy revealed non-necrotizing granulomatous inflammation suggestive of sarcoidosis. The neurologic deficits and orbital inflammation responded well to intravenous corticosteroids. Neurosarcoidosis can present with unusual clinical manifestations, as evidenced by this patient.

## Introduction

Sarcoidosis is an immune-mediated, non-caseating granulomatous inflammatory disorder that can present with varied multiorgan involvement [1]. Neurological involvement is evident in 3-15% of cases, with an estimated annual incidence of about 0.5 cases/100,000 population [2,3]. Symptomatic neurosarcoidosis classically manifests as cranial neuropathy and can also affect the meninges, ventricles, hypothalamic-pituitary axis, brainstem, cerebellum, and spinal cord [4]. Spinal cord involvement is rare, accounting for only 1% of neurosarcoidosis cases [5]. Spinal cord involvement presenting with orbital involvement and identified through an orbital biopsy is particularly rare.

Spinal neurosarcoidosis can manifest as cord atrophy, radiculopathy, syringomyelia, arachnoiditis, cauda equina syndrome, conus medullaris syndrome, and transverse myelitis [5,6]. Spinal cord involvement in sarcoidosis may be difficult to diagnose when there are no clear clinical signs pointing to the diagnosis. Neuroimaging of spinal sarcoidosis shows non-specific hyperintensity on magnetic resonance imaging (MRI) in the affected neural tissue. The ideal test for the diagnosis of neurosarcoidosis remains a biopsy of tissue, which poses logistical challenges [5].

## Case presentation

This 64-year-old woman with no significant past medical history reported experiencing intermittent tingling in her fingers beginning four months prior to her presentation. After two months of symptoms without resolution, the patient saw her primary care provider and was ultimately referred to neurology. She presented to neurology with numbness in her left hand and bilateral lower extremities following the development of flu-like symptoms. The patient denied any exposure to infection. Neither a respiratory pathogen panel nor a gastrointestinal pathogen panel was performed, but the meningitis pathogen panel returned negative. She described the lower extremity numbness as feeling like she was standing on cotton balls. The patient also reported a feeling of tightness around her chest and lower legs. She denied incontinence but reported incomplete control of urination and defecation. The numbness in her feet progressed, and she developed weakness, which created increasing difficulty with walking.

Outpatient MRI of the cervical and thoracic spinal cord was obtained, revealing diffuse signal abnormality and multifocal enhancement suspicious for acute myelitis. She was then sent to the emergency department for suspicion of acute transverse myelitis extending from spinal level C2 to T8 (image unavailable). The etiology was unclear, prompting further investigation. A lumbar puncture was performed. Flow cytometry revealed that, while her lymphocytes and monocytes were elevated, there was no evidence of clonal lymphoid expansion, and this process was likely reactive. Angiotensin-converting enzyme (ACE), myelin basic protein, oligoclonal bands, aquaporin-4 antibodies, myelin oligodendrocyte glycoprotein antibodies, and Lyme antibodies were all normal or negative. Paraneoplastic antibodies were negative. The presumptive diagnosis at this time was post-infectious transverse myelitis, for which she was hospitalized and treated with high-dose solumedrol 250 mg every six hours for 20 doses completed in the hospital. The patient was discharged on a tapering dose of prednisone starting at 60 mg daily, decreasing by 10 mg every fourth day, and followed up with a neurologist outpatient. While on steroid treatment, the patient saw mild improvements to her presenting symptoms but not full resolution. On discharge, she was a well-appearing woman who was alert and oriented to her surroundings. Her vitals were blood pressure of 142/72, pulse of 72 beats/min, 16 breaths/min, and temperature of 98.3 F. Her head was atraumatic and normocephalic. She had non-labored breathing and intact sensation in all four extremities. However, she left with a wide-based and unsteady gait. 

**Figure 1:**
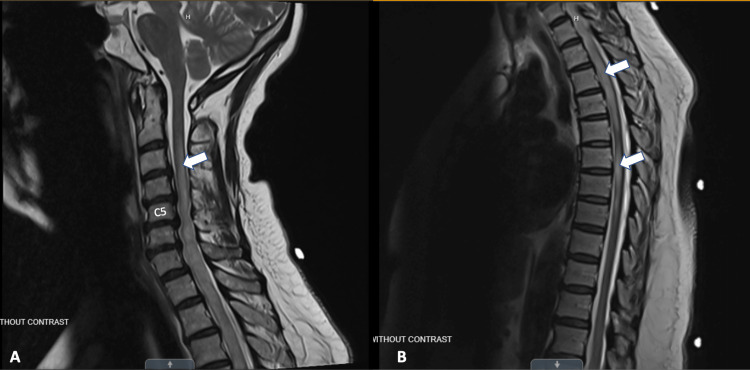
Cervical and thoracic T2 MRI. (A) T2 cervical spine MRI-transverse myelitis in the cervical region with the extension of cord signal abnormality (white arrow) with mass effect on the cervical cord at C5-C7 (C5 labeled on image) due to worsening cord swelling. (B) T2 thoracic spine MRI-areas of T2 bright signal (white arrows), cord expansion and cord enhancement in the thoracic region extending down to the T8 level. MRI: magnetic resonance imaging.

**Figure 2:**
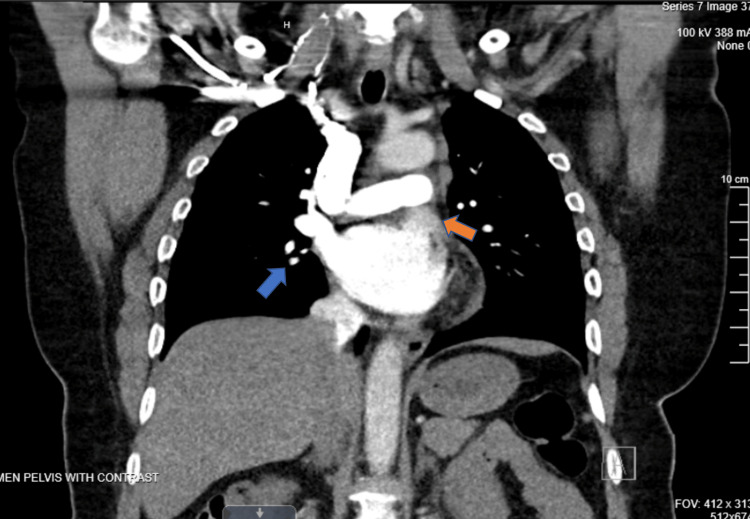
CT of the chest. Contrast-enhanced CT of the chest showing right hilar and mediastinal lymphadenopathy (orange arrow) and calcified subcarinal nodes (blue arrow). CT: computed tomography.

Three months after her presentation, she developed recurrent and increased difficulty walking due to weakness and tightness around her trunk that was not completely resolved from her previous presentation. Repeat cervical and thoracic MRI (Figures 1A, 1B) showed persistent areas of abnormal T2 hyperintensity and patchy enhancement throughout the cervical and thoracic spinal cord, which was minimally diminished. Computed tomography (CT) of the chest showed right hilar and mediastinal lymphadenopathy and calcified subcarinal nodes (Figure 2). During this hospitalization, she developed swelling of her left upper eyelid. She underwent plasmapheresis for seven days and was discharged home. On discharge, she was a well-appearing woman who was alert and oriented to her surroundings. No focal deficits were elicited. Her head was atraumatic and normocephalic. She had non-labored breathing and intact sensation in all four extremities. The patient reported subjective improvement in leg movement.

The patient presented to the hospital again two months later due to progressive weakness in her lower legs and diminished ability to ambulate. She complained of dragging her right leg and could not walk without support. She also stated that the tightness in her trunk has worsened. A mass was evident in her upper left upper eyelid medially. 

On this admission, she was a well-appearing woman who was alert and oriented to her surroundings. Her vitals were blood pressure of 130/89, pulse of 89 beats/min, 16 breaths/min, and temperature of 98 F. Mild periorbital swelling of the left eye was noted, but the patient denied changes to her vision. The patient did not report symptoms or have exam findings consistent with uveitis. She had non-labored breathing and intact sensation in all four extremities. She had 5/5 strength in her bilateral upper extremities, 2/5 strength in her right lower extremity, and 4/5 strength in her left lower extremity. As a biopsy of the patient’s left orbital mass was already scheduled, cerebrospinal fluid (CSF) was not obtained at this admission for analysis at the recommendation of the neurology team unless the workup remained unclear.

**Figure 3:**
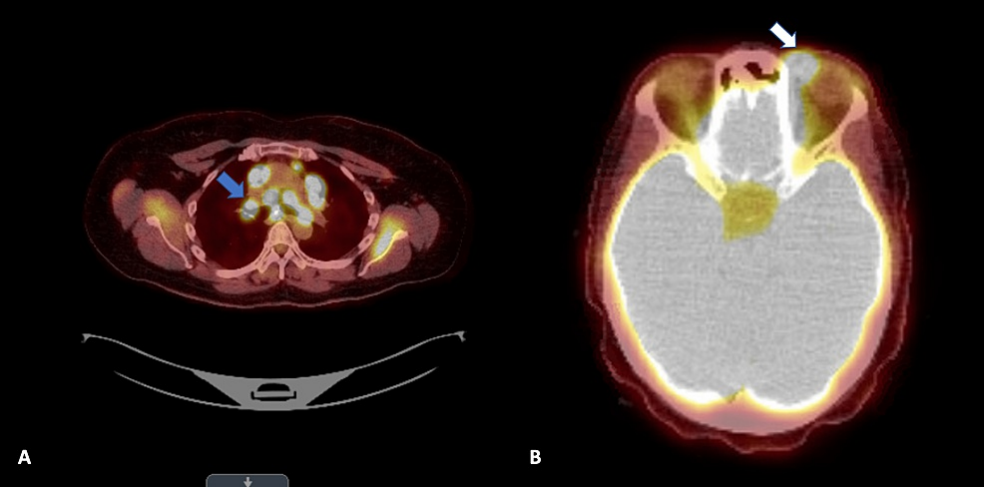
Positron emission tomography (PET) scan. (A) Mildly enlarged lymph nodes seen within the mediastinum as well as within the pulmonary hila, which display abnormal hypermetabolism (blue arrow). (B) Curvilinear focus of hypermetabolism in the medial left orbit (white arrow).

PET scan showed mildly enlarged lymph nodes seen within the mediastinum as well as within the pulmonary hila (Figure 3A). These findings correlated with the lymphadenopathy evident on a CT scan of the chest in July. These lymph nodes displayed abnormal hypermetabolism with a standardized uptake value (SUV) of 39. Below the diaphragm, mildly enlarged lymph nodes within the porta hepatis displayed abnormal hypermetabolism with SUV 8.2. Curvilinear focus of hypermetabolism in the medial left orbit corresponded to asymmetric soft tissue density on the CT scan (Figure 3B).

**Figure 4:**
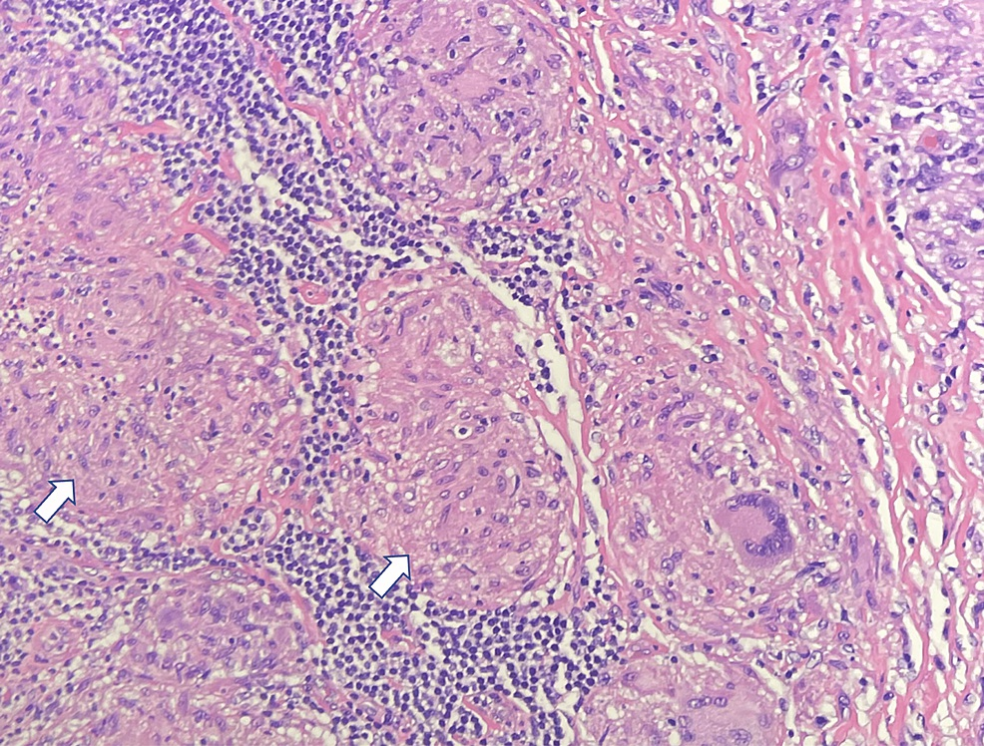
Histological section of orbital mass. Section of the orbital mass showing granulomas (white arrows) composed of epithelioid histiocytes and multinucleated giant cells associated with mild to moderate lymphocytic chronic inflammation.

Removal of the orbital mass was already scheduled prior to this admission for cosmetic reasons. Pulmonary deferred biopsy of the hilar lymphadenopathy unless the already scheduled workup remained unclear to avoid unnecessary invasive procedures. Incisional biopsy of the left orbital mass revealed non-necrotizing granulomatous inflammation suggestive of sarcoidosis (Figure 4). The patient was started on high-dose solumedrol 500 mg every 12 h for five days, then switched to prednisone 60 mg daily to taper. The patient was also started on hydroxychloroquine 200 mg daily. She was discharged to subacute rehab with follow-up with rheumatology and neurology.

## Discussion

Sarcoidosis can present with unusual symptoms, particularly neurosarcoidosis. In the patient presented in this case report, unexplained neurological symptoms and findings due to transverse myelitis had no clear cause until a synchronous diagnosis of orbital sarcoidosis was made. Less than 10% of sarcoidosis cases involve neurological symptoms, and among those, fewer than 10% involve non-cranial nerves [4,5,7]. The most common means of establishing the diagnosis of neurosarcoidosis is with a mediastinal biopsy [7]. In this case, the mediastinal disease was evident after orbital biopsy, and the initial clinical response to systemic corticosteroids masked the true diagnosis of neurosarcoidosis.

Effective treatment of neurosarcoidosis is important because uncontrolled chronic inflammation can cause permanent neurological damage [8]. Spontaneous remission of spinal neurosarcoidosis is uncommon, while post-infectious transverse myelitis is more commonly self-limited. Effective control of spinal disease due to neurosarcoidosis typically requires prolonged treatment. Spinal neurosarcoidosis is often more resistant to steroid treatment as compared to neurosarcoidosis with predominantly cranial involvement [4,9]. In extreme cases of spinal neurosarcoidosis where there is evidence of spinal cord compression, surgical decompression via granuloma resection may be needed [9].

## Conclusions

Sarcoidosis is a multisystem disease that is characterized by non-caseating granulomatous inflammation in affected organs. Neurosarcoidosis can have protean manifestations and can be difficult to recognize. We have described a case of neurosarcoidosis that presented as transverse myelitis and was diagnosed after extension to orbital sarcoidosis.
